# Amputation-specific and generic correlates of participation among Veterans with lower limb amputation

**DOI:** 10.1371/journal.pone.0270753

**Published:** 2022-07-07

**Authors:** Christopher R. Erbes, John Ferguson, Kalia Yang, Sara Koehler-McNicholas, Melissa A. Polusny, Brian J. Hafner, Allen W. Heinemann, Jessica Hill, Tonya Rich, Nicole Walker, Marilyn Weber, Andrew Hansen

**Affiliations:** 1 Center for Care Delivery and Outcomes Research, Minneapolis VA Healthcare System, Minneapolis, Minnesota, United States of America; 2 Department of Psychiatry and Behavioral Sciences, University of Minnesota Medical School, Minneapolis, Minnesota, United States of America; 3 Minneapolis Adaptive Design and Engineering Program, Minneapolis VA Healthcare System, Minneapolis, Minnesota, United States of America; 4 Department of Rehabilitation Medicine, University of Minnesota, Minneapolis, Minnesota, United States of America; 5 Departments of Rehabilitation Medicine and Bioengineering, University of Washington, Seattle, Washington, United States of America; 6 Departments of Physical Medicine and Rehabilitation, Emergency Medicine, and Medical Social Sciences, Feinberg School of Medicine, Northwestern University and Shirley Ryan AbilityLab, Chicago, Illinois, United States of America; 7 Department of Biomedical Engineering, University of Minnesota, Minneapolis, Minnesota, United States of America; University of Illinois at Chicago, UNITED STATES

## Abstract

Participation in valued interpersonal and community activities is a key component of rehabilitation for Veterans with amputation. The purpose of this study was to identify specific factors that promote or inhibit participation to inform development of interventions that may facilitate participation in desired life activities. A convenience sample of 408 Veterans with at least one lower limb amputation and who had received outpatient care from the Regional Amputation Center (RAC) completed a mailed survey. Participation was measured using the Community Participation Indicators (CPI) Importance, Control, and Frequency scales and the Patient Reported Outcome Measurement Information System (PROMIS) Ability to Participate in Social Roles and Satisfaction with Social Participation scales. Multiple imputation procedures were used to address missing data. Correlates of participation were examined through multiple linear regression. A total of 235 participants completed the survey, a response rate of 58%. Levels of participation, measured with the PROMIS instruments, were 43.2 (SD = 8.1) for Ability and 46.4 (SD = 8.6) for Satisfaction. Regression analyses found robust amputation-specific correlates for participation, including body image and balance confidence. Generic (non-amputation specific) correlates for participation included depression and pain interference. Development of treatment approaches and devices that can address body image, balance confidence, pain, and mental health concerns such as depression have the potential to enhance the participation and rehabilitation of Veterans with lower limb amputation.

## Introduction

The United States Departments of Veterans Affairs (VA) and Defense (DOD) offer comprehensive acute and rehabilitative care for service members and Veterans with amputation. These services have been integrated into systems of care such as the Amputee Patient Care Program in the DOD [1] and the Amputation System of Care in the VA [2]. Despite the impressive array of services and technologies available to today’s Veterans, treatment programs generally focus more on improving physical function than on evaluating and enhancing social and community participation (i.e., reintegration) [3]. Successful rehabilitation also requires re-engagement and participation in a range of personal, family, and social roles. Participation has been acknowledged as a key component of successful rehabilitation from severe medical or mental health conditions [4]. Thus, the development and analysis of treatment approaches for Veterans living with amputation may benefit from an improved understanding of Veterans’ participation in preferred life roles and activities.

Following the World Health Organization’s International Classification of Functioning, Disability and Health (ICF) model [5], we view successful rehabilitation as extending beyond body functions and physical activity performance to include functioning in a social context (i.e., participation). While studies of Veterans with lower limb amputation have focused on a range of outcomes including physical capabilities [6], depression and mental health [7, 8], and overall quality of life [6, 9, 10], fewer have focused specifically on participation in necessary or important life activities. Early investigations found individuals with amputations had good social and job participation outcomes, but rated lower in other areas related to participation, such as community mobility, physical functioning, and recreation [11, 12]. More recently, Roepke and colleagues reported that participation in social activities among Veterans with lower limb amputations was associated with baseline mental status, physical health, and amputation level (with more distal amputations correlating to greater participation), while satisfaction with social participation was associated with baseline social support [13]. The study also highlighted the distinction between satisfaction with participation and frequency of participation and suggested that both are important rehabilitation targets. The broader literature on quality of life also suggests potential correlates of participation, including mental health (e.g., depression [7, 9, 14]), level and type of amputation [6], social support [8], prosthesis use and satisfaction [10], and medical comorbidity [7, 10, 14].

The ICF model presents functioning across three domains (body function, activity, and participation) as being determined by physical conditions, environmental factors (outside of the individual), and personal factors. The development of rehabilitation interventions may further be bolstered by distinguishing between physical, environmental, and personal factors that are generic (meaning applicable regardless of the particular health condition being studied) and amputation-specific (meaning those with particular pertinence due to the problems and adaptations entailed with amputation). By distinguishing the factors that contribute to participation into generic and amputation-specific considerations, we may be able to tailor interventions to the needs of the individual (e.g., generic factors may be served with mental health interventions whereas amputation-specific factors may be addressed with rehabilitation or prosthesis interventions).

The purpose of this study was to identify specific factors that promote or inhibit participation to inform future development of interventions that may facilitate participation in desired life activities. Generic factors that might be linked with participation in the general Veteran population were included: 1) physical (general pain intensity and interference; medical comorbidity), 2) social support, and 3) mental health symptoms (posttraumatic stress disorder [PTSD] symptoms, depression, and anxiety, given their high prevalence among Veterans and potential impact on participation [15, 16]). Amputation-specific factors thought to associate with participation included those related to physical health (e.g., residual limb health and pain, phantom limb pain), as well as perceived mobility, balance confidence, body image, and prosthesis utility. Amputation-specific factors were those that were most relevant to the amputation rehabilitation team and that could be affected through provision of or changes to a prosthesis, medication (for amputation-specific pain), or other physical rehabilitation interventions (e.g., physical therapy). We hypothesized that amputation-specific factors would add uniquely to the statistical prediction of participation when controlling for generic factors. In keeping with recent conceptualizations of participation [13], we used multiple indices to assess perceived ability to participate in social roles, satisfaction with participation, frequency of activities performed, and the perceived importance and control over such activities.

## Methods

### Procedures

A cross-sectional survey was administered to a convenience sample of Veterans with lower limb amputation to address the study aims. We identified potential participants using two methods, the 1) VA electronic medical record and 2) Minneapolis VA Healthcare System’s Regional Amputation Center (RAC) amputation patient database. Eligibility criteria included unilateral or bilateral lower limb amputation, and current receipt of amputation care from the Minneapolis VA RAC. Veterans completed a standardized survey assessing a number of patient-reported outcomes collected in the summer of 2016 using standard mailed survey methodology [17]. Two weeks after sending a survey pre-notification letter, participants were mailed a cover letter containing the elements of informed consent and survey packet. At 2-week intervals, a post-card reminder and two additional survey mailings were sent to non-respondents. Up to six reminder calls were used to enhance response rates and maximize representativeness of our convenience sample. Respondents received $20 compensation for returning a completed survey. The protocol was approved by the Minneapolis VA Health Care System Institutional Review Board.

### Measures

Members of our multidisciplinary research team identified several measures to explore the influence of amputation-specific and generic factors on participation in a sample of Veterans with amputations. The cross-sectional survey included multiple patient-reported outcome measures selected to measure the domains of interest: physical and personal factors; social support; mental health symptoms; and amputation factors. Three of these domains included Patient Reported Outcome Measures Information System (PROMIS) instruments. PROMIS short forms are fixed-length instruments, developed from PROMIS item banks, that provide T scores (mean = 50, SD = 10). The PROMIS measures included in this study allow for comparison to a normative sample from the U.S. general population of healthy adults. The included PROMIS pain measures (PROMIS-Pain Interference, PROMIS Pain Intensity) also include pain items used to calibrate the data from the normative sample of the U.S. general population to a clinical sample of individuals with chronic pain [18].

#### Physical and personal factors

Care Assessment Needs Index 2.0 (CAN 2.0) [19]. The second edition of the VA’s CAN 2.0 score was used as an index of medical comorbidity and disease burden. The CAN 2.0 score is derived from the VA’s electronic medical records database using an algorithm designed to predict patient outcomes including hospitalization or death within a given time frame. The CAN 2.0 algorithm incorporates 36 variables including age, body mass index, heart rate, prior hospitalizations and types of hospitalizations, lab visits, and the Deyo-Charlson comorbidity score [20]. The 90-Day Event score, indexing the probability of a hospitalization in the next 90 days, was used in the present study. Previous studies have indicated a relationship between the presence of comorbidities and quality-of-life among those with lower limb amputations, and we anticipate comorbidity status may also influence participation [9].

Community Participation Indicators (CPI) [21, 22]. The perceived personal value and importance of a person’s current community activities was assessed with the 14-item Importance Subscale of the CPI (CPI-Importance). Perceptions of control and efficacy over community activities were assessed with the 13-item Control subscale (CPI-Control). Scores for each subscale range from 0 to 100 and are based on normative data from a large sample of people with a range of health conditions. A frequency index was derived by taking the mean of 20 items asking about the frequency of activities over the past week (CPI-Frequency). Items ranged from “Get out and about” to “Exercise, participate in sports or active recreation,” and were rated from 0 (none in the last week) to 6 (5 or more times). The CPI-Importance and CPI-Control subscales have been shown to vary inversely with disability severity [22]. The CPI-Frequency has not been used in prior studies but provides a metric of general engagement in community-based activities.

Demographic Variables. We collected demographic characteristics (i.e., age, race, marital status, amputation type and level (i.e., bilateral versus unilateral, at or above knee versus below knee), and occupational status within our printed survey. Participants were asked about amputations on each side and bilateral amputation was defined as amputations (at any level including toes or partial foot) on both sides. Participants having multiple amputations were coded with their higher level of amputation (e.g., someone having below-knee and above-knee amputations would be coded with an amputation level of above-knee).

Pain Interference. We used the 8-item PROMIS Pain Interference 8a (PROMIS-PI) short form to assess perceived limitations due to pain and the 3-item PROMIS Pain Intensity 3a (PROMIS-Pain Intensity) short form to assess pain intensity [23, 24].

Social Role Participation and Satisfaction. Two PROMIS measures were used to assess participation. We used the PROMIS Short Form v2.0—Ability to Participate in Social Roles and Activities 8a (PROMIS-Participation) and the PROMIS Short Form v2.0—Satisfaction with Social Roles and Activities 4a (PROMIS-Satisfaction) [25, 26]. The PROMIS-Participation is part of the PROMIS-29 v2 and the PROMIS-Satisfaction is part of the PROMIS-29 v1 battery that was originally designed for use across a range of clinical populations. Prior reports suggest excellent internal consistency in other chronic clinical populations for the PROMIS-Participation (0.93, 0.90) and PROMIS-Satisfaction (0.96 and 0.90) [27]. Prior reports of the PROMIS-Satisfaction in individuals with lower limb amputation also suggest worse satisfaction with social roles as compared to normative data [28].

#### Social support

Multidimensional Scale of Perceived Social Support (MSP) [25]. The 12-item MSP was used to measure perceived functional social support from three sources: Family, Friends, and Significant Others (loosely defined as a “special person”). Scale scores range from 1 to 7, with higher scores reflecting greater support. The MSP has good internal reliability and strong factorial validity [29]. Prior work in amputation populations suggests that the MSP is a predictor of pain interference, life satisfaction, and mobility [30].

Instrumental Social Support. Instrumental social support (e.g., perceived availability for active support in day-to-day tasks such as transportation, cooking, or housework) was assessed with the 8-item PROMIS-Instrumental Support v2.0 8a (PROMIS-IS) short-form [26]. The PROMIS-IS provides the perceived availability of social support with use of this measure to examine other aging populations [31].

#### Mental health symptoms

Anxiety and Depression [32]. Mental health symptoms were assessed with the 8-item PROMIS Anxiety 8a (PROMIS-Anxiety) and the 8-item PROMIS Depression 8a (PROMIS-Depression) short forms [32]. Prior work in the PROMIS-Anxiety and PROMIS-Depression in the Veteran population suggest Veterans report worse scores as compared to the general US population norms [33]. Data from PROMIS instruments in a recent study suggests that depression and anxiety are associated with quality of life in individuals with lower limb amputation [34].

Primary Care PTSD Screen (PC-PTSD) [35]. The PC-PTSD was given as a brief screen for the presence of PTSD symptomatology. The PC-PTSD has 4 items and provides a total score of 0 to 4, where higher scores indicate greater post-traumatic distress. The PC-PTSD had good test-retest reliability in the primary care population [35].

#### Amputation factors

Activities-specific Balance Confidence (ABC) [36]. The ABC is 16-item measure of balance confidence (efficacy) that has shown good convergent and known-groups construct validity among people with lower limb amputations [37]. We administered the ABC using a condition-specific, 5-point ordinal response scale, with higher scores reflecting greater confidence [38]; this amputation-specific version has demonstrated excellent test-retest reliability (0.95) among users of lower-limb prostheses [39]. Although the ABC has been used in populations beyond those with lower limb amputation, it was selected as amputation-specific for our analysis as low scores could be addressed by the amputation rehabilitation team through changes to the prosthesis or therapy.

Amputee Body Image Scale–Revised (ABIS-R) [40]. The ABIS-R uses 14 items to assess concerns with body image based on a person’s amputation and/or prosthesis. Responses are on a 1 to 3 scale, with 3 indicating greater difficulties. The total score has been shown to correlate inversely with measures of social adjustment and activity and positively with social restriction. The ABIS-R has good internal consistency (Cronbach’s alpha of 0.90) in the lower limb amputation population [40].

Prosthetic Limb Users Survey of Mobility (PLUS-M) [41]. The 12-item PLUS-M short form (Version 1.2) was used to measure perceived mobility. The PLUS-M provides a T score centered on a large national sample of individuals with lower limb amputation. The PLUS-M has demonstrated excellent concurrent and known-groups validity [42]. The 12-item PLUS-M short form also has been reported to have excellent test-retest reliability (0.96) in lower limb prosthesis users [39].

Prosthesis Evaluation Questionnaire (PEQ) [43]. Several subscales of the PEQ were used to assess prosthesis- and amputation-specific health concerns. The 8-item Prosthesis Utility scale evaluates prosthetic characteristics such as comfort, fit, and ease of use. The 6-item Residual Limb Health scale assesses physical difficulties related to prosthesis use such as rashes, sores, or sweating. In addition, two questions about the frequency and duration of specific pains were averaged to provide indices of phantom limb pain and residual limb pain. Similar to other studies, we used a 7-point ordinal response scale instead of the original visual analog scale to ease respondent burden [44]. All of the PEQ scales are reported to have high internal consistency [43].

### Analysis

We based our approach on social science approaches, such as those described by Tabachnick and Fidell [45]. First, all proposed variables were examined for significant correlations. Variable distributions were examined using frequency analyses. Multiple imputation procedures were executed using the MICE package in R in order to avoid the bias that occurs with data that are not missing completely at random, and to avoid a decrease in statistical power [46]. In multiple imputation, statistics were run with each imputed data set and then results were pooled across data sets taking into account variability within and between imputed data [47]. Ten complete datasets were imputed with Markhov Chain Monte Carlo estimation based on all study indicators, as well as age, race, marital status, amputation type (bilateral versus unilateral, at or above knee versus below knee), and occupational status. Although regression strategies identify independent indicators of dependent variables, we will refer to independent variables in this study as correlates.

We used multiple linear regression to identify independent associations between the primary outcome variable participation and other variables (correlates) with a fixed (*a priori)* block entry to avoid overfitting the data set. Each participation variable was regressed on the set of potential correlates in two stages. First, general correlates were entered as a block, followed by amputation-specific correlates as a second block. Block 1 included participant age. Block 2 added amputation at or above knee and double amputation variables. Finally, Block 3 added PROMIS Anxiety, Depression, Pain Interference, Pain Intensity, and Instrumental Support variables. We examined change in R-squared to determine how much variance in the dependent variable was accounted for by the set of independent variables at each step. Beta coefficients were used to determine the extent of prediction for each independent variable. We considered a more stringent criteria of adjusting for all comparisons (i.e., the test of each coefficient in each regression), but this would have resulted in an overly stringent correction, requiring p-values of less than 0.05/(18 x 5 = 90) = 0.0005. Therefore, we incorporated a Bonferroni adjustment with a p-value of 0.01 or less with a family-wise error of 0.05 overall. Standard linear regression assumptions (i.e., homoscedasticity, multicollinearity) were assessed. Homoscedasticity was assessed by examination of scatter plots, while the absence of multicollinearity was assessed using the Variance Inflation Factors (VIF), with values over 10 suggesting the presence of multicollinearity.

## Results

### Participants

Review of patient records identified 473 individuals who met the study inclusion criteria. After excluding 65 deceased individuals confirmed via chart review or returned mail, a pool of 408 eligible participants was identified. A final sample of 235 Veterans returned surveys (58% response rate). The characteristics of this sample are provided in Table 1.

**Table 1:** Sample demographics (N = 235).

Characteristic		n		%	Mean (SD), range
Race					
	White/Caucasian	210		89.4%	
	American Indian	10		4.3%	
	African American	6		2.6%	
	Did Not Report		7	3.0%	
Sex					
	Male	232		98.7%	
	Female	3		1.3%	
Relationship Status					
	Married	127		54.0%	
	In relationship, cohabitating	18		7.7%	
	In relationship, not cohabitating	8		3.4%	
	Single	68		28.9%	
	Did not report	14		6.0%	
Education (highest level)					
	Some high school or diploma/GED	89		37.9%	
	Come college or Associate’s degree	86		36.6%	
	Bachelor’s degree	27		11.5%	
	Graduate degree	14		6.0%	
	Did not report	19		8.1%	
Employment					
	Retired or unemployed	191		81.3%	
	Working or students	29		12.3%	
	Did not report	15		6.4%	
Amputation Characteristics					
	Unilateral	176		74.9%	
	Bilateral	44		18.7%	
	Below the knee	151		64.3%	
	At or above the knee (at least one)	69		29.4%	
	Did not report	15		6.4%	
Prosthesis Use					
	Never	14		6.0%	
	Only once or twice	1		0.4%	
	A few times	3		1.3%	
	Fairly often	5		2.1%	
	Very often	7		3.0%	
	Several times every day	6		2.6%	
	All or almost all the time	156		66.4%	
	Did not report	43		18.3%	
Age					64.1 (11.4), 24–94

### Participation and its correlates

No data were missing for 163 participants (69.4%). Across study variables, data were missing from 0% to 12.8% of cases, with a mean level of missingness of 5.5%. The only variable missing more than 10% was the PEQ Prosthesis Utility subscale (12.8%); participants who expressed they never used a prosthesis (n = 14) were instructed to skip the PEQ Prosthesis Utility subscale, which may account for some of this missingness. Analyses were run using both the complete (with list-wise deletion, n = 163) and the imputed (using pooled values, n = 235) data sets. For the most part, results were consistent across approaches, with some increase in power evident in the imputed data. Pooled (imputed) results are reported below.

Descriptive statistics, bivariate correlations of participation indices and potential correlates, and internal consistencies of each specific measure in the present sample are provided in Tables 2 and 3. Overall, participation variables were moderately to highly correlated, with the strongest correlations between PROMIS-Participation and PROMIS-Satisfaction (r = 0.72, p < 0.001) and CPI-Control and Importance (r = 0.70, p < 0.001). On average, participants reported diminished ability to participate in social roles and activities (mean PROMIS-Participation T-score = 43.2 (SD = 8.1)) and satisfaction with their participation (mean PROMIS-Satisfaction T-score = 46.4 (SD = 8.6)).

**Table 2:** Descriptive statistics for study variables.

	α	Mean	Minimum	Maximum	SD
CPI Importance	.92	43.83	0.00	100.00	11.59
CPI Control	.93	59.17	0.00	100.00	13.70
CPI Frequency	.77	1.23	0.10	3.20	0.50
PROMIS Ability	.95	43.20	25.90	65.40	8.05
PROMIS Satisfaction	.92	46.42	28.40	62.80	8.62
PROMIS Pain intensity	.90	47.95	30.70	71.80	8.78
PROMIS Pain interference	.96	57.40	40.70	77.00	8.67
PC-PTSD	.83	1.08	0.00	4.00	1.45
PROMIS Anxiety	.95	50.29	37.10	78.00	9.76
PROMIS Depression	.95	51.16	38.20	81.10	9.58
PROMIS Instrumental Support	.96	54.48	27.00	65.60	9.95
MSP Support—Friend	.94	5.02	1.00	7.00	1.48
MSP Support—Family	.93	5.38	1.00	7.00	1.58
MSP Support—Sig. Other	.94	5.57	1.00	7.00	1.67
CAN 2.0 Score	---	70.24	0.00	99.00	24.23
PEQ Residual Limb Pain	.80	2.45	0.00	6.00	1.66
PEQ Phantom Limb Pain	.69	2.45	0.00	6.00	1.52
PEQ Residual Limb Health	.77	2.64	0.17	4.00	0.86
PEQ Prosthesis Utility	.87	2.54	0.50	4.00	0.81
PLUS-M Mobility	.96	44.67	21.80	71.40	11.28
ABC Balance Confidence	.96	2.06	0.06	4.00	0.99
ABIS-R Body Image	.89	9.99	0.00	28.00	6.38

Notes. Activities-specific Balance Confidence (ABC), Amputee Body Image Scale–Revised (ABIS-R), Care Assessment Needs Index 2.0 (CAN 2.0), Community Participation Indicators (CPI), Multidimensional Scale of Perceived Social Support (MSP), Patient Reported Outcome Measurement Information System (PROMIS), Primary Care PTSD Screen (PC-PTSD), Prosthesis Evaluation Questionnaire (PEQ), and Prosthetic Limb Users Survey of Mobility (PLUS-M).

α = Cronbach’s alpha (internal consistency within the items of each individual measure) in the present sample. A Cronbach’s alpha of 0.70 is acceptable and 0.80 or greater is preferred.

* p < .05

** p < .01

*** p < .001

**Table 3:** Correlations of participation indicators with predictors.

	Importance		Control		Frequency		Ability		Satisfaction	
CPI Importance	1.00		0.70	***	0.64	***	0.49	***	0.58	***
CPI Control			1.00		0.53	***	0.56	***	0.68	***
CPI Frequency					1.00		0.42	***	0.47	***
PROMIS Ability							1.00		0.72	***
PROMIS Satisfaction									1.00	
Race (African-American)	-0.26	***	-0.17	*	-0.17	*	-0.15	*	-0.06	
Age	-0.07		0.07		-0.07		0.02		0.03	
Double amputation	-0.01		-0.05		0.09		-0.12		-0.10	
Above knee amputation	0.02		0.06		0.03		0.04		0.06	
Partnered	0.09		0.05		0.22	**	-0.01		0.03	
Any college	0.19	**	0.12		0.08		0.11		0.08	
Working	0.14	*	0.02		0.16	*	0.16	*	0.16	*
PROMIS Pain intensity	-0.18	**	-0.21	**	-0.10		-0.22	**	-0.20	**
PROMIS Pain interference	-0.29	***	-0.34	***	-0.17	*	-0.34	***	-0.31	***
PC-PTSD	-0.16	*	-0.16	*	-0.14	*	-0.15	*	-0.18	**
PROMIS Anxiety	-0.12		-0.31	***	-0.14	*	-0.18	**	-0.30	***
PROMIS Depression	-0.37	***	-0.43	***	-0.31	***	-0.32	***	-0.45	***
PROMIS Instrumental Support	0.19	**	0.22	***	0.25	***	0.05		0.19	**
MSP Support—Friend	0.37	***	0.38	***	0.36	***	0.23	***	0.36	***
MSP Support–Family	0.17	**	0.24	***	0.26	***	0.10		0.19	**
MSP Support—Sig. Other	0.13		0.23	***	0.27	***	0.04		0.14	*
CAN 2.0 Score	-0.20	**	-0.18	**	-0.21	**	-0.22	**	-0.27	***
PEQ Residual Limb Pain	-0.11		-0.20	**	-0.04		-0.14	*	-0.18	**
PEQ Phantom Limb Pain	-0.12		-0.20	**	-0.06		-0.15	*	-0.19	**
PEQ Residual Limb Health	0.16	*	0.26	***	0.00		0.28	***	0.29	***
PEQ Prosthesis Utility	0.35	***	0.39	***	0.29	***	0.43	***	0.51	***
PLUS-M Mobility	0.34	***	0.33	***	0.35	***	0.52	***	0.49	***
ABC Balance Confidence	0.39	***	0.42	***	0.43	***	0.59	***	0.58	***
ABIS-R Body Image	-0.43	***	-0.48	***	-0.29	***	-0.55	***	-0.50	***

Notes. Activities-specific Balance Confidence (ABC), Amputee Body Image Scale–Revised (ABIS-R), Care Assessment Needs Index 2.0 (CAN 2.0), Community Participation Indicators (CPI), Multidimensional Scale of Perceived Social Support (MSP), Patient Reported Outcome Measurement Information System (PROMIS), Primary Care PTSD Screen (PC-PTSD), Prosthesis Evaluation Questionnaire (PEQ), and Prosthetic Limb Users Survey of Mobility (PLUS-M).

* p < .05

** p < .01

*** p < .001

The CPI-Frequency is a measure of frequency of activity rather than an appraisal of activity. As expected, the CPI-Frequency was weakly correlated with other participation indicators (ranging from 0.41 to 0.64). For the most part, demographic variables, and those relating to the characteristics of the amputation, were not significantly correlated with participation. However, participants with partners reported greater participation frequency (CPI-Frequency r = 0.22, p = 0.001), and those with at least some college education reported more perceived importance of activities (CPI-Importance r = 0.19, p = 0.005). Working or attending school was associated positively with most participation outcomes (r ranged from 0.14 to 0.16), with the exception of CPI-Control (r = 0.02). Finally, African-American race was associated with diminished perceived ability (PROMIS-Ability, r = -0.15), CPI-Importance (r = -0.26), CPI-Control (r = -0.17), and CPI-Frequency (r = -0.17). Thus, we included African-American race as a covariate in all regression models given its statistical correlation with these participation indices.

Results of these multiple linear regression models are summarized in Tables 4–8. Prior to conducting multiple linear regression, the standard linear regression assumptions were assessed. An examination of residual and scatter plots indicated the assumptions of normality, linearity, and homoscedasticity were all met. Collinearity statistics (i.e., Tolerance and VIF) were all within accepted limits indicating the assumption of the absence of multicollinearity was deemed to have been met. Model performance was evaluated by interpreting R^2^ values, which were above .2 for all models. The general factors (Block 1) had strong associations with each participation indicator (R^2^ from 0.22 to 0.32) and the amputation-specific factors (Block 2) added incrementally and significantly to that association (R^2^ Δ from 0.10 to 0.35). A portion of variables in Block 1 (4 of 11 variables) and Block 2 (1 of 7 variables) were independently associated with CPI-Importance. The PROMIS-Depression score was associated with all participation measures, and pain interference was associated with all but the CPI-Frequency score. Support from a friend was associated with all but the PROMIS-Participation score, and medical comorbidity (CAN 2.0 score) was associated with both PROMIS-Participation and PROMIS-Satisfaction measures, as well as CPI-Frequency. Among amputation-specific factors, body image was associated with both PROMIS-Participation and PROMIS-Satisfaction participation measures, as well as CPI- Importance and Control. Balance confidence was associated with both PROMIS-Participation and PROMIS-Satisfaction measures and CPI-Frequency.

**Table 4:** Regression of CPI importance on general and specific indicators.

Independent Variable	B	SE(B)	Beta	t	p
Block 1 (General Predictors)^1^					
Intercept	59.62	7.37	0.00	8.09	0.000
Race (African-American)	-14.35	4.29	-0.20	-3.35	0.001
PROMIS Pain Intensity	0.07	0.12	0.05	0.54	0.588
PROMIS Pain Interference	-0.24	0.12	-0.18	-1.98	0.049
PC-PTSD PTSD	0.45	0.60	0.06	0.74	0.460
PROMIS Anxiety	0.23	0.09	0.19	2.44	0.016
PROMIS Depression	-0.47	0.11	-0.39	-4.31	0.000
PROMIS Support—Instrumental	0.13	0.08	0.11	1.56	0.119
MSP Support—Friend	1.89	0.58	0.24	3.25	0.001
MSP Support—Family	-0.75	0.61	-0.10	-1.22	0.223
MSP Support—Sig. Other	-0.29	0.59	-0.04	-0.49	0.622
CAN 2.0 Score	-0.05	0.03	-0.11	-1.83	0.069
Block 2 (Amputation Specific)^2^					
PEQ Residual Limb Pain	-0.24	0.54	-0.03	-0.45	0.655
PEQ Phantom Limb Pain	0.59	0.47	0.08	1.25	0.214
PEQ Residual Limb Health	-0.35	0.90	-0.03	-0.39	0.695
PEQ Prosthesis Utility	1.53	1.23	0.11	1.24	0.215
PLUS-M Mobility	-0.01	0.11	-0.01	-0.12	0.908
ABC Balance Confidence	1.65	1.25	0.14	1.32	0.188
ABIS-R Body Image	-0.49	0.13	-0.27	-3.84	0.000

Notes. Activities-specific Balance Confidence (ABC), Amputee Body Image Scale–Revised (ABIS-R), Care Assessment Needs Index 2.0 (CAN 2.0), Community Participation Indicators (CPI), Multidimensional Scale of Perceived Social Support (MSP), Patient Reported Outcome Measurement Information System (PROMIS), Primary Care PTSD Screen (PC-PTSD), Prosthesis Evaluation Questionnaire (PEQ), and Prosthetic Limb Users Survey of Mobility (PLUS-M).

Block 1 coefficients displayed are unadjusted for Block 2 indicators in the model.

^1^ R^2^ = .32, Wald (df = 11, 12555.18) = 5.28, p < .001

^2^ Δ R_2_ = .11, Wald (df = 7, 2420.65) = 2.74, p = .008

**Table 5:** Regression of CPI control on general and specific indicators.

Independent Variable	B	SE(B)	Beta	t	p
Block 1 (General Predictors)^1^					
Intercept	87.33	8.89	0.00	9.82	0.000
Race (African-American)	-7.68	5.03	-0.09	-1.52	0.129
PROMIS Pain Intensity	0.09	0.14	0.05	0.61	0.543
PROMIS Pain Interference	-0.35	0.14	-0.22	-2.41	0.017
PC-PTSD PTSD	1.30	0.67	0.14	1.94	0.053
PROMIS Anxiety	-0.11	0.11	-0.08	-1.04	0.301
PROMIS Depression	-0.40	0.12	-0.28	-3.29	0.001
PROMIS Support—Instrumental	0.11	0.10	0.08	1.14	0.256
MSP Support—Friend	2.00	0.72	0.22	2.76	0.006
MSP Support—Family	-0.47	0.73	-0.05	-0.64	0.525
MSP Support—Sig. Other	0.55	0.68	0.07	0.81	0.418
CAN 2.0 Score	-0.05	0.03	-0.09	-1.55	0.123
Block 2 (Amputation Specific)^2^					
PEQ Residual Limb Pain	-0.43	0.59	-0.05	-0.73	0.467
PEQ Phantom Limb Pain	-0.07	0.56	-0.01	-0.12	0.907
PEQ Residual Limb Health	0.99	1.08	0.06	0.92	0.361
PEQ Prosthesis Utility	0.64	1.38	0.04	0.46	0.646
PLUS-M Mobility	0.00	0.13	0.00	0.00	0.997
ABC Balance Confidence	2.71	1.46	0.20	1.86	0.065
ABIS-R Body Image	-0.47	0.15	-0.22	-3.12	0.002

Notes. Activities-specific Balance Confidence (ABC), Amputee Body Image Scale–Revised (ABIS-R), Care Assessment Needs Index 2.0 (CAN 2.0), Community Participation Indicators (CPI), Multidimensional Scale of Perceived Social Support (MSP), Patient Reported Outcome Measurement Information System (PROMIS), Primary Care PTSD Screen (PC-PTSD), Prosthesis Evaluation Questionnaire (PEQ), and Prosthetic Limb Users Survey of Mobility (PLUS-M).

Block 1 coefficients displayed are unadjusted for Block 2 indicators in the model.

^1^ R^2^ = .32, Wald (df = 11, 14984.60) = 3.38, p < .001

^2^ Δ R_2_ = .12, Wald (df = 7, 2458.81) = 2.10, p = .041

**Table 6:** Regression of CPI frequency on general and specific indicators.

Independent Variable	B	SE(B)	Beta	t	p
Block 1 (General Predictors)^1^					
Intercept	1.29	0.36	0.00	3.63	0.000
Race (African-American)	-0.32	0.20	-0.10	-1.56	0.121
PROMIS Pain Intensity	0.00	0.01	0.05	0.56	0.576
PROMIS Pain Interference	0.00	0.01	-0.07	-0.69	0.489
PC-PTSD PTSD	0.01	0.03	0.03	0.37	0.715
PROMIS Anxiety	0.00	0.00	0.08	0.98	0.326
PROMIS Depression	-0.01	0.00	-0.27	-2.94	0.004
PROMIS Support—Instrumental	0.01	0.00	0.11	1.49	0.138
MSP Support—Friend	0.06	0.03	0.18	2.25	0.026
MSP Support—Family	-0.01	0.03	-0.04	-0.41	0.685
MSP Support—Sig. Other	0.03	0.03	0.10	1.15	0.250
CAN 2.0 Score	0.00	0.00	-0.15	-2.14	0.033
Block 2 (Amputation Specific)^2^					
PEQ Residual Limb Pain	-0.02	0.02	-0.08	-0.95	0.344
PEQ Phantom Limb Pain	0.03	0.02	0.09	1.35	0.178
PEQ Residual Limb Health	-0.08	0.05	-0.13	-1.74	0.084
PEQ Prosthesis Utility	0.03	0.05	0.04	0.48	0.631
PLUS-M Mobility	0.00	0.01	-0.02	-0.19	0.852
ABC Balance Confidence	0.16	0.06	0.31	2.62	0.010
ABIS-R Body Image	-0.01	0.01	-0.10	-1.14	0.257

Notes. Activities-specific Balance Confidence (ABC), Amputee Body Image Scale–Revised (ABIS-R), Care Assessment Needs Index 2.0 (CAN 2.0), Community Participation Indicators (CPI), Multidimensional Scale of Perceived Social Support (MSP), Patient Reported Outcome Measurement Information System (PROMIS), Primary Care PTSD Screen (PC-PTSD), Prosthesis Evaluation Questionnaire (PEQ), and Prosthetic Limb Users Survey of Mobility (PLUS-M).

Block 1 coefficients displayed are unadjusted for Block 2 indicators in the model.

^1^ R^2^ = .24, Wald (df = 11, 10319.83) = 2.4, p = .006

^2^ Δ R_2_ = .10, Wald (df = 7, 1531.31) = 2.03, p = .049

**Table 7:** Regression of PROMIS ability to participate on general and specific indicators.

Independent Variable	B	SE(B)	Beta	t	p
Block 1 (General Predictors)^1^					
Intercept	65.82	5.66	0.00	11.63	0.000
Race (African-American)	-4.94	3.20	-0.10	-1.54	0.124
PROMIS Pain Intensity	0.06	0.09	0.07	0.71	0.480
PROMIS Pain Interference	-0.27	0.10	-0.29	-2.80	0.006
PC-PTSD PTSD	0.39	0.43	0.07	0.90	0.368
PROMIS Anxiety	0.05	0.07	0.06	0.75	0.455
PROMIS Depression	-0.21	0.08	-0.25	-2.73	0.007
PROMIS Support—Instrumental	-0.01	0.06	-0.01	-0.19	0.849
MSP Support—Friend	0.75	0.46	0.14	1.64	0.103
MSP Support—Family	-0.26	0.47	-0.05	-0.56	0.578
MSP Support—Sig. Other	-0.14	0.44	-0.03	-0.32	0.752
CAN 2.0 Score	-0.05	0.02	-0.15	-2.31	0.022
Block 2 (Amputation Specific)^2^					
PEQ Residual Limb Pain	-0.28	0.32	-0.06	-0.90	0.369
PEQ Phantom Limb Pain	0.51	0.30	0.10	1.73	0.086
PEQ Residual Limb Health	1.05	0.56	0.11	1.85	0.065
PEQ Prosthesis Utility	-0.33	0.69	-0.03	-0.47	0.637
PLUS-M Mobility	0.08	0.07	0.12	1.27	0.206
ABC Balance Confidence	2.97	0.77	0.37	3.86	0.000
ABIS-R Body Image	-0.44	0.09	-0.35	-4.89	0.000

Notes. Activities-specific Balance Confidence (ABC), Amputee Body Image Scale–Revised (ABIS-R), Care Assessment Needs Index 2.0 (CAN 2.0), Community Participation Indicators (CPI), Multidimensional Scale of Perceived Social Support (MSP), Patient Reported Outcome Measurement Information System (PROMIS), Primary Care PTSD Screen (PC-PTSD), Prosthesis Evaluation Questionnaire (PEQ), and Prosthetic Limb Users Survey of Mobility (PLUS-M).

Block 1 coefficients displayed are unadjusted for Block 2 indicators in the model.

^1^ R^2^ = .22, Wald (df = 11, 8961.45) = 2.58, p = .003

^2^ Δ R_2_ = .35, Wald (df = 7, 1761.93) = 7.57, p < .001

**Table 8:** Regression of PROMIS satisfaction on general and specific indicators.

Independent Variable	B	SE(B)	Beta	t	p
Block 1 (General Predictors)^1^					
Intercept	63.91	5.55	0.00	11.51	0.000
Race (African-American)	1.26	3.18	0.02	0.40	0.692
PROMIS Pain Intensity	0.09	0.09	0.09	1.07	0.284
PROMIS Pain Interference	-0.19	0.09	-0.19	-2.16	0.032
PC-PTSD PTSD	0.56	0.42	0.09	1.32	0.188
PROMIS Anxiety	-0.06	0.07	-0.06	-0.78	0.438
PROMIS Depression	-0.26	0.08	-0.29	-3.35	0.001
PROMIS Support—Instrumental	0.09	0.06	0.10	1.44	0.151
MSP Support—Friend	1.50	0.46	0.26	3.28	0.001
MSP Support—Family	-0.15	0.46	-0.03	-0.32	0.752
MSP Support—Sig. Other	-0.36	0.44	-0.07	-0.82	0.411
CAN 2.0 Score	-0.07	0.02	-0.19	-3.21	0.002
Block 2 (Amputation Specific)^2^					
PEQ Residual Limb Pain	-0.38	0.33	-0.07	-1.14	0.258
PEQ Phantom Limb Pain	0.17	0.34	0.03	0.50	0.616
PEQ Residual Limb Health	0.76	0.62	0.08	1.22	0.223
PEQ Prosthesis Utility	1.28	0.79	0.12	1.62	0.107
PLUS-M Mobility	0.04	0.07	0.06	0.68	0.500
ABC Balance Confidence	2.60	0.80	0.30	3.27	0.001
ABIS-R Body Image	-0.28	0.08	-0.21	-3.41	0.001

Notes. Activities-specific Balance Confidence (ABC), Amputee Body Image Scale–Revised (ABIS-R), Care Assessment Needs Index 2.0 (CAN 2.0), Community Participation Indicators (CPI), Multidimensional Scale of Perceived Social Support (MSP), Patient Reported Outcome Measurement Information System (PROMIS), Primary Care PTSD Screen (PC-PTSD), Prosthesis Evaluation Questionnaire (PEQ), and Prosthetic Limb Users Survey of Mobility (PLUS-M).

Block 1 coefficients displayed are unadjusted for Block 2 indicators in the model.

^1^ R^2^ = .32, Wald (df = 11, 14819.79) = 4.02, p < .001

^2^ Δ R_2_ = .25, Wald (df = 7, 2264.04) = 3.97, p < .001

## Discussion

Results of this study support the importance of both generic and, as hypothesized, amputation-specific correlates of participation among Veterans with lower limb amputation. Satisfaction with social roles was similar to the level reported by Amtmann and colleagues [28] in their national survey of 1,028 adults with lower limb amputation (PROMIS-Satisfaction T-score = 48.0). Perceptions of control over activities (CPI-Control score = 59.2) and the importance of those activities (43.8) were mixed. The higher scores on CPI-Control may reflect that the full CPI was normed on a group of individuals with different physical disabilities rather than a U.S. general population sample [21]. Characteristics of the amputation (e.g., unilateral vs. bilateral or amputation level) were not robustly related to participation. This finding may reflect the difference between the indicators of physical functioning that are often studied and appraisals of community and interpersonal participation [28, 48].

In our regression model, depression was the most consistent general independent correlate, being significantly associated with all five participation indicators. This reinforces that, although some studies do not find elevated rates of depression among populations with amputation, mental health screening and intervention remain important components for rehabilitation efforts [7, 28]. Chronic pain also requires attention, as pain interference often is elevated in this population and it was a significant independent correlate of importance and control of activities, as well as satisfaction and perceived ability to participate in social roles [28, 49]. Of the social support variables examined, only support from friends emerged as a significant independent correlate of the participation variables (importance, control, frequency, and ability). This finding, in conjunction with studies showing that social support is related to pain interference, life satisfaction, and mobility among persons with lower limb amputation [50], suggests that psychosocial interventions that enhance social connections and support, and that extend beyond immediate family members, may also be important.

Of the amputation-specific correlates, amputation-specific body image was independently associated with importance and control over community participation as well as perceived satisfaction and ability. Thus, attention to psychological health, physical appearance of the prosthesis and of the whole person as related to the amputation may be important facilitators of community participation. This finding complements previous assertions that psychological intervention focused on body image and self-esteem may positively influence these factors among those with lower extremity amputations [51]. This also corroborates prior findings in individuals with amputation who indicated the ability (or inability) to choose the proper clothing or footwear for an activity as a significant contributor to the experience of a “good” or “bad” day [52]. Prostheses that allow for a fuller range of clothing options, including footwear, may be important for Veterans. As our sample was predominantly male, it will be important for future studies to assess this relationship in female Veterans with amputation. Prostheses that allow a natural gait may also be helpful in providing a positive body image and may lead to improved participation. Our findings are consistent with those of Miller et al. [53] and Sions et al. [54] in demonstrating the importance of balance confidence for community participation. Prostheses and therapy interventions that improve balance performance and balance confidence may have positive effects on participation.

This study is a first step toward understanding which variables are most correlated with participation. Use of these measures with those for participation may be helpful to amputation rehabilitation teams and other interdisciplinary medical teams toward addressing amputation-specific and generic factors that associate with participation. Future studies should also examine the effects of targeted interventions on the correlates of participation to see if they lead to improvements in participation of persons with lower limb amputations.

### Study limitations

Our results are limited in that they come from a self-selected, local convenience sample that may not represent the Veteran population as a whole in terms of geographic and demographic characteristics. Nonresponse bias is always present in postal-based surveys, and it may be that those who did respond to our survey had different levels of participation compared to those who did not respond. This study did not assess amputation characteristics (e.g., etiology and time since amputation), and therefore could not assess or build upon literature on the association of these characteristics with Veteran social and community participation.

The cross-sectional study design precludes causal conclusions. Thus, our statistical correlates may in fact be reciprocally related to participation, or even caused by it, rather than the other way around. However, our use of multiple indices of participation allows an examination of both levels of activity (frequency) as well as subjective appraisals of that activity, and our use of a wide range of correlates helps identify specific targets for intervention that contribute to participation in the Veteran population.

Our study results may not generalize to non-Veterans as the potential exists that access to medical care, including provision of prostheses, is greater within the VA Healthcare System. Additionally, it is unknown if the presence and severity of comorbidities are comparable between individuals with amputations depending on Veteran status. Future research in larger, non-Veteran samples could examine the generalizability of our findings.

This study was not sufficiently powered to evaluate the influence of demographic factors on participation. However, racial disparities regarding amputation risk exist, with individuals of African American, Native American, and Hispanic backgrounds being at significantly greater risk of lower extremity amputation than Caucasian individuals [55, 56]. African-American race was included in our regression analyses due to its statistical correlation with included indices of participation. However, future studies including diverse samples and measures of multiple levels of influence (e.g., person-, social-, system-, and societal-level) are needed to explore the implications of the relationship between participation and demographic factors.

Lastly, our survey included factors thought by our team to correlate with participation; however, many other factors not measured in our study may also correlate with participation (e.g., prosthesis satisfaction, motivation, prosthesis wear time). Future work is needed to gather qualitative data from Veterans with amputations to identify other factors associated with participation. Ideally this work would be ongoing to continuously identify and remove barriers for successful participation.

## Conclusions

Veterans with lower limb amputation showed reduced levels of perceived ability and satisfaction with their social role participation relative to national norms. Depression, pain, body image and balance confidence were correlated independently with participation measures. The development of treatment approaches and devices that address these factors remains an important priority.

## Supporting information

S1 Data (XLSX)

S1 Table Regression of CPI importance on general and specific indicators, listwise deletion (N = 163). (DOCX)

S2 Table Regression of CPI control on general and specific indicators (N = 163). (DOCX)

S3 Table Regression of CPI frequency on general and specific indicators (N = 163). (DOCX)

S4 Table Regression of PROMIS ability to participate on general and specific indicators (N = 163). (DOCX)

S5 Table Regression of PROMIS satisfaction on general and specific indicators (N = 163). (DOCX)

## List of abbreviations

ABC: Activities-specific Balance Confidence Scale
ABIS-R: Amputee Body Image Scale—Revised
CAN 2.0: Care Assessment Needs Index Version 2.0
CPI: Community Participation Indicators
CPI: Control Community Participation Indicators-Control subscale
CPI-Frequency: Community Participation Indicators-Frequency Index
CPI-Importance: Community Participation Indicators-Importance subscale
DOD: Department of Defense
ICF: International Classification of Functioning, Disability, and Health
MSP: Multidimensional Scale of Perceived Social Support
PC-PTSD: Primary Care Posttraumatic Stress Disorder Screen
PEQ: Prosthesis Evaluation Questionnaire
PLUS-M: Prosthetic Limb Users Survey of Mobility
PROMIS: Patient Reported Outcome Measurement Information System
PROMIS-Anxiety: Patient Reported Outcome Measurement Information System Short Form v1.0—Anxiety 8a
PROMIS-Depression: Patient Reported Outcome Measurement Information System Short Form v1.0—Depression 8a
PROMIS-IS: Patient Reported Outcome Measurement Information System Short Form v2.0—Instrumental Support 8a
PROMIS-Pain Intensity: Patient Reported Outcome Measurement Information System Scale v2.0—Pain Intensity 3a
PROMIS-PI: Patient Reported Outcome Measurement Information System Short Form v1.1—Pain Interference 8a
PROMIS-Participation: Patient Reported Outcome Measurement Information System Short Form v2.0—Ability to Participate in Social Roles and Activities 8a
PROMIS-Satisfaction: Patient Reported Outcome Measurement Information System Short Form v2.0—Satisfaction with Social Roles and Activities 4a
RAC: Regional Amputation Center
VA: Department of Veterans Affairs
