# Supplementary material for: Amputation-specific and generic correlates of participation among Veterans with lower limb amputation
Source: PLoS One. 2022 Jul 7;17(7):e0270753. doi: 10.1371/journal.pone.0270753 (PMC9262244; doi:10.1371/journal.pone.0270753)
Supplement: S1 Table — (DOCX) [file pone.0270753.s002.docx]

S1 Table. Regression of CPI Importance on General and Specific Indicators, Listwise Deletion (N = 163)

| Independent Variable | B | SE(B) | Beta | t | p |
| --- | --- | --- | --- | --- | --- |
| Block 1 (General Predictors)^1^ |  | |  |  |  |
| Intercept | 57.23 | 9.42 |  | 6.07 | 0.000 |
| Race (African-American) | -11.76 | 5.16 | -0.16 | -2.28 | 0.024 |
| PROMIS Pain Intensity | 0.14 | 0.14 | 0.10 | 1.00 | 0.317 |
| PROMIS Pain Interference | -0.26 | 0.14 | -0.19 | -1.92 | 0.056 |
| PC-PTSD PTSD | -0.04 | 0.65 | -0.01 | -0.06 | 0.949 |
| PROMIS Anxiety | 0.13 | 0.11 | 0.11 | 1.21 | 0.229 |
| PROMIS Depression | -0.38 | 0.12 | -0.32 | -3.17 | 0.002 |
| PROMIS Support - Instrumental | 0.18 | 0.10 | 0.16 | 1.89 | 0.061 |
| MSP Support - Friend | 2.72 | 0.74 | 0.34 | 3.65 | 0.000 |
| MSP Support - Family | -1.17 | 0.70 | -0.16 | -1.68 | 0.094 |
| MSP Support - Sig. Other | -1.01 | 0.73 | -0.14 | -1.39 | 0.166 |
| CAN 2.0 Score | -0.05 | 0.03 | -0.10 | -1.34 | 0.181 |
| Block 2 (Amputation Specific)^2^ |  | |  | | |
| PEQ Residual Limb Pain | -0.27 | 0.61 | -0.04 | -0.44 | 0.658 |
| PEQ Phantom Limb Pain | 0.77 | 0.54 | 0.10 | 1.42 | 0.158 |
| PEQ Residual Limb Health | -0.69 | 1.07 | -0.05 | -0.64 | 0.520 |
| PEQ Prosthesis Utility | 0.48 | 1.36 | 0.03 | 0.35 | 0.728 |
| PLUS-M Mobility | 0.04 | 0.15 | 0.03 | 0.26 | 0.797 |
| ABC Balance Confidence | 1.81 | 1.59 | 0.15 | 1.14 | 0.257 |
| ABIS-R Body Image | -0.47 | 0.16 | -0.26 | -2.93 | 0.004 |

Notes. Activities-specific Balance Confidence (ABC), Amputee Body Image Scale – Revised (ABIS-R), Care Assessment Needs Index 2.0 (CAN 2.0), Community Participation Indicators (CPI), Multidimensional Scale of Perceived Social Support (MSP), Patient Reported Outcome Measurement Information System (PROMIS), Primary Care PTSD Screen (PC-PTSD), Prosthesis Evaluation Questionnaire (PEQ), and Prosthetic Limb Users Survey of Mobility (PLUS-M).

Block 1 coefficients displayed are unadjusted for Block 2 indicators in the model.

^1^ R^2^ = .35, F[11,151] = 7.23, p < .001

^2^ ∆R^2^ = .10, F[7,144] = 3.55, p = .002
