# Supplementary material for: Amputation-specific and generic correlates of participation among Veterans with lower limb amputation
Source: PLoS One. 2022 Jul 7;17(7):e0270753. doi: 10.1371/journal.pone.0270753 (PMC9262244; doi:10.1371/journal.pone.0270753)
Supplement: S2 Table — (DOCX) [file pone.0270753.s003.docx]

S2 Table. Regression of CPI Control on General and Specific indicators (N = 163)

| Independent Variable | B | SE(B) | Beta | t | p |
| --- | --- | --- | --- | --- | --- |
| Block 1 (General Predictors)^1^ |  | |  |  |  |
| Intercept | 80.90 | 10.05 |  | 8.05 | 0.000 |
| Race (African-American) | -7.09 | 5.50 | -0.09 | -1.29 | 0.199 |
| PROMIS Pain Intensity | 0.17 | 0.15 | 0.11 | 1.15 | 0.253 |
| PROMIS Pain Interference | -0.34 | 0.15 | -0.22 | -2.32 | 0.022 |
| PC-PTSD PTSD | 0.43 | 0.70 | 0.05 | 0.61 | 0.541 |
| PROMIS Anxiety | -0.25 | 0.12 | -0.19 | -2.10 | 0.038 |
| PROMIS Depression | -0.30 | 0.13 | -0.23 | -2.36 | 0.020 |
| PROMIS Support - Instrumental | 0.19 | 0.10 | 0.14 | 1.78 | 0.077 |
| MSP Support - Friend | 1.75 | 0.79 | 0.20 | 2.20 | 0.029 |
| MSP Support - Family | -0.20 | 0.74 | -0.02 | -0.27 | 0.789 |
| MSP Support - Sig. Other | 0.41 | 0.78 | 0.05 | 0.53 | 0.594 |
| CAN 2.0 Score | -0.04 | 0.04 | -0.08 | -1.15 | 0.252 |
| Block 2 (Amputation Specific)^2^ |  | |  | | |
| PEQ Residual Limb Pain | 0.29 | 0.64 | 0.04 | 0.44 | 0.657 |
| PEQ Phantom Limb Pain | 0.01 | 0.57 | 0.00 | 0.02 | 0.986 |
| PEQ Residual Limb Health | 1.88 | 1.12 | 0.12 | 1.68 | 0.095 |
| PEQ Prosthesis Utility | 1.18 | 1.43 | 0.07 | 0.83 | 0.410 |
| PLUS-M Mobility | 0.15 | 0.15 | 0.12 | 0.99 | 0.323 |
| ABC Balance Confidence | 1.42 | 1.67 | 0.11 | 0.85 | 0.396 |
| ABIS-R Body Image | -0.23 | 0.17 | -0.11 | -1.38 | 0.168 |

Notes. Activities-specific Balance Confidence (ABC), Amputee Body Image Scale – Revised (ABIS-R), Care Assessment Needs Index 2.0 (CAN 2.0), Community Participation Indicators (CPI), Multidimensional Scale of Perceived Social Support (MSP), Patient Reported Outcome Measurement Information System (PROMIS), Primary Care PTSD Screen (PC-PTSD), Prosthesis Evaluation Questionnaire (PEQ), and Prosthetic Limb Users Survey of Mobility (PLUS-M).

Block 1 coefficients displayed are unadjusted for Block 2 indicators in the model.

^1^ R2 = .40, F[11,151] = 9.01, p < .001

^2^ ∆ R2 = .11, F[7,144] = 4.31, p < .001
