# Supplementary material for: Amputation-specific and generic correlates of participation among Veterans with lower limb amputation
Source: PLoS One. 2022 Jul 7;17(7):e0270753. doi: 10.1371/journal.pone.0270753 (PMC9262244; doi:10.1371/journal.pone.0270753)
Supplement: S3 Table — (DOCX) [file pone.0270753.s004.docx]

S3 Table. Regression of CPI Frequency on General and Specific Indicators (N = 163)

| Independent Variable | B | SE(B) | Beta | t | p |
| --- | --- | --- | --- | --- | --- |
| Block 1 (General Predictors)^1^ |  | |  |  |  |
| Intercept | 1.14 | 0.44 |  | 2.56 | 0.011 |
| Race (African-American) | -0.26 | 0.24 | -0.08 | -1.09 | 0.278 |
| PROMIS Pain Intensity | 0.01 | 0.01 | 0.08 | 0.81 | 0.418 |
| PROMIS Pain Interference | 0.00 | 0.01 | -0.06 | -0.59 | 0.554 |
| PC-PTSD PTSD | 0.01 | 0.03 | 0.02 | 0.25 | 0.803 |
| PROMIS Anxiety | 0.00 | 0.01 | 0.09 | 0.86 | 0.389 |
| PROMIS Depression | -0.01 | 0.01 | -0.27 | -2.52 | 0.013 |
| PROMIS Support - Instrumental | 0.01 | 0.00 | 0.21 | 2.29 | 0.024 |
| MSP Support - Friend | 0.07 | 0.04 | 0.19 | 1.92 | 0.057 |
| MSP Support - Family | -0.01 | 0.03 | -0.03 | -0.31 | 0.757 |
| MSP Support - Sig. Other | 0.00 | 0.03 | 0.00 | 0.00 | 0.998 |
| CAN 2.0 Score | 0.00 | 0.00 | -0.19 | -2.55 | 0.012 |
| Block 2 (Amputation Specific)^2^ |  | |  | | |
| PEQ Residual Limb Pain | -0.24 | 0.69 | -0.03 | -0.36 | 0.724 |
| PEQ Phantom Limb Pain | 0.87 | 0.58 | 0.11 | 1.48 | 0.141 |
| PEQ Residual Limb Health | -1.22 | 1.15 | -0.09 | -1.07 | 0.289 |
| PEQ Prosthesis Utility | 0.68 | 1.39 | 0.04 | 0.49 | 0.626 |
| PLUS-M Mobility | 0.00 | 0.15 | 0.00 | 0.02 | 0.985 |
| ABC Balance Confidence | 2.33 | 1.66 | 0.20 | 1.41 | 0.162 |
| ABIS-R Body Image | -0.48 | 0.16 | -0.26 | -0.30 | 0.004 |

Notes. Activities-specific Balance Confidence (ABC), Amputee Body Image Scale – Revised (ABIS-R), Care Assessment Needs Index 2.0 (CAN 2.0), Community Participation Indicators (CPI), Multidimensional Scale of Perceived Social Support (MSP), Patient Reported Outcome Measurement Information System (PROMIS), Primary Care PTSD Screen (PC-PTSD), Prosthesis Evaluation Questionnaire (PEQ), and Prosthetic Limb Users Survey of Mobility (PLUS-M).

Block 1 coefficients displayed are unadjusted for Block 2 indicators in the model.

^1^ R^2^ = .25, F[11,151] = 4.61, p < .001

^2^ ∆ R^2^ = .09, F[7,144] = 2.91, p = .007
