# Supplementary material for: Amputation-specific and generic correlates of participation among Veterans with lower limb amputation
Source: PLoS One. 2022 Jul 7;17(7):e0270753. doi: 10.1371/journal.pone.0270753 (PMC9262244; doi:10.1371/journal.pone.0270753)
Supplement: S4 Table — (DOCX) [file pone.0270753.s005.docx]

S4 Table. Regression of PROMIS Ability to Participate on General and Specific Indicators (N = 163)

| Independent Variable | B | SE(B) | Beta | t | p |
| --- | --- | --- | --- | --- | --- |
| Block 1 (General Predictors)^1^ |  | |  |  |  |
| Intercept | 63.35 | 7.02 |  | 9.03 | 0.000 |
| Race (African-American) | -1.63 | 3.84 | -0.03 | -0.42 | 0.672 |
| PROMIS Pain Intensity | 0.10 | 0.10 | 0.10 | 0.97 | 0.331 |
| PROMIS Pain Interference | -0.25 | 0.10 | -0.25 | -2.42 | 0.017 |
| PC-PTSD | -0.33 | 0.49 | -0.06 | -0.68 | 0.499 |
| PROMIS Anxiety | 0.00 | 0.08 | 0.00 | 0.02 | 0.988 |
| PROMIS Depression | -0.13 | 0.09 | -0.16 | -1.48 | 0.141 |
| PROMIS Support - Instrumental | 0.05 | 0.07 | 0.06 | 0.62 | 0.534 |
| MSP Support - Friend | 0.90 | 0.55 | 0.16 | 1.63 | 0.106 |
| MSP Support - Family | -0.38 | 0.52 | -0.07 | -0.73 | 0.465 |
| MSP Support - Sig. Other | -0.73 | 0.54 | -0.14 | -1.35 | 0.180 |
| CAN 2.0 Score | -0.06 | 0.03 | -0.19 | -2.41 | 0.017 |
| Block 2 (Amputation Specific)^2^ |  | |  | | |
| PEQ Residual Limb Pain | -0.10 | 0.35 | -0.02 | -0.30 | 0.768 |
| PEQ Phantom Limb Pain | 0.65 | 0.31 | 0.13 | 2.10 | 0.037 |
| PEQ Residual Limb Health | 0.89 | 0.61 | 0.09 | 1.46 | 0.145 |
| PEQ Prosthesis Utility | 0.86 | 0.78 | 0.08 | 1.10 | 0.271 |
| PLUS-M Mobility | 0.18 | 0.08 | 0.23 | 2.15 | 0.033 |
| ABC Balance Confidence | 1.85 | 0.91 | 0.22 | 2.02 | 0.045 |
| ABIS-R Body Image | -0.43 | 0.09 | -0.35 | -4.73 | 0.000 |

Notes. Activities-specific Balance Confidence (ABC), Amputee Body Image Scale – Revised (ABIS-R), Care Assessment Needs Index 2.0 (CAN 2.0), Community Participation Indicators (CPI), Multidimensional Scale of Perceived Social Support (MSP), Patient Reported Outcome Measurement Information System (PROMIS), Primary Care PTSD Screen (PC-PTSD), Prosthesis Evaluation Questionnaire (PEQ), and Prosthetic Limb Users Survey of Mobility (PLUS-M).

Block 1 coefficients displayed are unadjusted for Block 2 indicators in the model.

^1^ R^2^ = .23, F[11,151] = 4.12, p < .001

^2^ ∆ R^2^ = .38, F[7,144] = 20.12, p < .001
