# Supplementary material for: Amputation-specific and generic correlates of participation among Veterans with lower limb amputation
Source: PLoS One. 2022 Jul 7;17(7):e0270753. doi: 10.1371/journal.pone.0270753 (PMC9262244; doi:10.1371/journal.pone.0270753)
Supplement: S5 Table — (DOCX) [file pone.0270753.s006.docx]

S5 Table. Regression of PROMIS Satisfaction on General and Specific Indicators (N = 163)

| Independent Variable | B | SE(B) | Beta | t | p |
| --- | --- | --- | --- | --- | --- |
| Block 1 (General Predictors)^1^ |  | |  |  |  |
| Intercept | 59.44 | 7.07 |  | 8.41 | 0.000 |
| Race (African-American) | 2.22 | 3.87 | 0.04 | 0.57 | 0.567 |
| PROMIS Pain Intensity | 0.18 | 0.10 | 0.17 | 1.81 | 0.073 |
| PROMIS Pain Interference | -0.21 | 0.10 | -0.20 | -2.09 | 0.039 |
| PC-PTSD PTSD | 0.10 | 0.49 | 0.02 | 0.20 | 0.841 |
| PROMIS Anxiety | -0.14 | 0.08 | -0.15 | -1.70 | 0.090 |
| PROMIS Depression | -0.20 | 0.09 | -0.22 | -2.22 | 0.028 |
| PROMIS Support - Instrumental | 0.16 | 0.07 | 0.18 | 2.14 | 0.034 |
| MSP Support - Friend | 1.76 | 0.56 | 0.28 | 3.16 | 0.002 |
| MSP Support - Family | -0.23 | 0.52 | -0.04 | -0.44 | 0.658 |
| MSP Support - Sig. Other | -0.62 | 0.55 | -0.11 | -1.14 | 0.257 |
| CAN 2.0 Score | -0.07 | 0.03 | -0.19 | -2.71 | 0.008 |
| Block 2 (Amputation Specific)^2^ |  | |  | | |
| PEQ Residual Limb Pain | -0.10 | 0.40 | -0.02 | -0.26 | 0.795 |
| PEQ Phantom Limb Pain | 0.25 | 0.35 | 0.04 | 0.71 | 0.477 |
| PEQ Residual Limb Health | 0.62 | 0.69 | 0.06 | 0.89 | 0.375 |
| PEQ Prosthesis Utility | 1.86 | 0.89 | 0.16 | 2.09 | 0.038 |
| PLUS-M Mobility | 0.11 | 0.09 | 0.13 | 1.18 | 0.240 |
| ABC Balance Confidence | 1.67 | 1.04 | 0.18 | 1.61 | 0.110 |
| ABIS-R Body Image | -0.31 | 0.10 | -0.22 | -3.00 | 0.003 |

Notes. Activities-specific Balance Confidence (ABC), Amputee Body Image Scale – Revised (ABIS-R), Care Assessment Needs Index 2.0 (CAN 2.0), Community Participation Indicators (CPI), Multidimensional Scale of Perceived Social Support (MSP), Patient Reported Outcome Measurement Information System (PROMIS), Primary Care PTSD Screen (PC-PTSD), Prosthesis Evaluation Questionnaire (PEQ), and Prosthetic Limb Users Survey of Mobility (PLUS-M).

Block 1 coefficients displayed are unadjusted for Block 2 indicators in the model.

^1^ 1 R^2^ = .38, F[11,151] = 8.38, p < .001

^2^ ∆ R^2^ = .22, F[7,144] = 12.09, p < .001
